# Supplementary material for: Determinants of the Essential Elements and Vitamins Intake and Status during Pregnancy: A Descriptive Study in Polish Mother and Child Cohort
Source: Nutrients. 2021 Mar 16;13(3):949. doi: 10.3390/nu13030949 (PMC8001522; doi:10.3390/nu13030949)
Supplement: Supplementary file 1 [file nutrients-13-00949-s001.pdf]

Determinants of the Essential Elements and Vitamins Intake and Status during Pregnancy: a Descriptive Study in Polish Mother and Child Cohort

Jankowska et al.

Supplementary materials

Table S1. Estimated average requirement (EAR) for selected essential elements and vitamins during pregnancy

Essential elements or vitamins	Pregnant women 1st trimester EAR
Calcium (mg/day)	800
Magnesium (mg/day)	300
Iron (mg/day)	23
Zinc (mg/day)	9.5
Copper (mg/day)	0.8
Selenium (µg/day)	50
Folate (µg/day)*	520
Vitamin A (µg/day)	530
Vitamin C (mg/day	100
Vitamin E (mg/day)	10
Vitamin B_1 (mg/day)	1.2
Vitamin B_2 (mg/day)	1.2
Vitamin B_3 (mg/day)	14
Vitamin B_6 (mg/day)	1.6
Vitamin B_{12} (µg/day)	2.2

Source: Jarosz M. Nutrition standards for Polish population. National Food and Nutrition Institute, Warsaw 2017. Available online: https://ncez.pl (accessed on 20 of December 2020)
* µg equivalent of folates

Table S2. Characteristics of the study population (N=1252)

Variables	Category	n (%)
**Sociodemographic**		
Maternal age (years)		
	17-30	799 (63.8)
	>30	431 (34.4)
	No data	22 (1.8)
Marital status		
	Married	1010 (80.7)
	Unmarried	231 (18.5)
	No data	11 (0.9)
Maternal education (years of education)		
	≤9	29 (2.3)
	10-12	345 (27.6)
	>12	867 (69.3)
	No data	11 (0.9)
Occupational activity between the 8th-12th week of pregnancy		
	No	492 (39.3)
	Yes	694 (55.4)
	No data	66 (5.3)
Socio-economic status (SES)		
	Low	90 (7.2)
	Medium	833 (66.5)
	High	301 (24.1)
	No data	28 (2.2)
**Lifestyle/health behavior**		
Pre-pregnancy BMI (kg/m^2)		
	<18.5	112 (8.9)
	18.5-24.99	891 (71.2)
	≥25	230 (18.4)
	No data	19 (1.5)
Cotinine level		
	< 10ng/ml	1110 (88.7)
	≥ 10ng/ml	139 (11.1)
	No data	3 (0.2)
Alcohol consumption		
	No	1154 (92.2)
	Yes	82 (6.5)
	No data	16 (1.3)
Leisure – time physical activity (LTPA)	No	387 (30.9)
	Yes	862 (68.9)
	No data	3 (0.2)
Perceived Stress Scale (range: 0-38 points) (PSS)		
	<17 points	589 (47.0)
	≥17 points	654 (52.3)
	No data	9 (0.7)
**Environmental**		
Place of residence (thousands of inhabitants)		
	<100	441 (35.2)
	≥100	809 (64.6)
	No data	2 (0.2)

Season for data collection		
	November – April	663 (53.0)
	May - October	589 (47.0)
**Pregnancy-related**		
Parity		
	0	639 (51.0)
	≥1	606 (48.4)
	No data	7 (0.6)
Adverse pregnancy symptoms and complications		
	No	1208 (96.5)
	Yes	44 (3.5)
	No data	0 (0.0)
Week of pregnancy of the 1st medical - care visit		
	≤6	739 (59.0)
	>6	492 (39.3)
	No data	21 (1.7)
Sex of the child		
	Male	565 (45.1)
	Female	539 (43.1)
	No data	148 (11.8)

Table S3. Percentage of pregnant women using dietary supplements and percentages of selected essential elements and vitamins from the supplements (n=1233)

Parameter	Percentage of pregnant women (%)*
Use of supplements during pregnancy	93.6
Calcium (mg/day)	10.6
Magnesium (mg/day)	12.4
Iron (mg/day)	23.6
Zinc (mg/day)	11.2
Copper (mg/day)	6.6
Selenium (µg/day)	17.8
Folate (µg/day)	99.8
Vitamin A (µg/day)	11.7
Vitamin C (mg/day)	41.1
Vitamin E (mg/day)	10.1
Vitamin B_1 (mg/day)	2.3
Vitamin B_2 (mg/day)	2.3
Vitamin B_3 (mg/day)	35.5
Vitamin B_6 (mg/day)	50.1
Vitamin B_{12} (µg/day)	38.5

*percentages for specific essential elements and vitamins calculated among the women taking supplements.

Table S4. Average daily intake of essential elements and vitamins with diet and in total (diet and supplements) (n = 1252)

Microelements /vitamins	Intake with diet<br>Median (range)	Intake with diet and supplements<br>Median (range)
Calcium (mg/day)	617.34 (119.18 - 2832.48)	624.74 (119.18 -2832.48)
Magnesium (mg/day)	238.50 (115.92 - 661.34)	246.75 (115.92 – 661.34)
Iron (mg/day)	9.89 (4.48 – 23.03)	10.80 (4.48 – 250.16)
Zinc (mg/day)	8.36 (4.05 – 23.12)	8.58 (4.11 – 165.50)
Copper (mg/day)	0.97 (0.42 – 2.94)	0.99 (0.42 – 4.30)
Selenium (µg/day)	54.29 (21.28 – 140.40)	57.27 (21.28 – 268.74)
Folate (µg/day)	250,32 (20.01 – 188.63)	1047.08 (133.17 – 5138.07)
Vitamin A (µg/day)	890.34 (211.50 – 5936.75)	944.67 (211.50 – 14193.68)
Vitamin C (mg/day)	90.98 (11.75 – 433.85)	123.73 (11,75 – 1214.74)
Vitamin E (mg/day)	8.47 (2.50 – 23.44)	8.74 (2.50 – 206.52)
Vitamin B_1 (mg/day)	1.10 (0.51 – 3.85)	1.10 (0.51 – 4.13)
Vitamin B_2 (mg/day)	1.55 (0.52 – 4.15)	1.55 (0.52 – 4.85)
Vitamin B_3 (mg/day)	16.05 (5.40 – 61.04)	18.95 (6.88 – 61.04)
Vitamin B_6 (mg/day)	1.73 (0.73 – 6.04)	2.50 (0.78 – 22.23)
Vitamin B_{12} (µg/day)	3.74 (0.89 – 26.41)	4.83 (1.09 – 26.41)

Table S5. Univariable models for adequacy of intake of selected essential elements and vitamins from dietary sources (n=1011)

Determinant	Calcium	Magnesium	Zinc	Copper	Selenium	Vitamin A	Vitamin C	Vitamin E	Vitamin B_1	Vitamin B_2	Vitamin B_3	Vitamin B_6	Vitamin B_{12}
							OR (95%CI)						
**Maternal age**													
> 30	1.01 (0.76, 1.33)	1.03 (0.76, 1.39)	1.16 (0.90, 1.50)	1.52 (1.14,2.05)*	1.65 (1.29,2.13)#	2.03 (1.34,3.18)*	0.97 (0.76, 1.23)	1.12 (0.86, 1.46)	1.01 (0.80, 1.29)	1.28 (0.96, 1.71)	1.60 (1.23, 2.10)*	1.07 (0.84, 1.36)	1.07 (0.72, 1.62)
**Maternal education (years of education)**													
> 12	1.44 (1.07,1.97)^	1.72 (1.23,2.43)*	1.39 (1.06,1.84)^	1.26 (0.95, 1.67)	1.38 (1.07,1.76)^	1.57 (1.08,2.27)^	1.37 (1.07,1.76)^	0.91 (0.69, 1.19)	1.31 (1.02,1.70)^	1.24 (0.93, 1.65)	1.41 (1.08, 1.82)^	1.15 (0.90, 1.47)	1.22 (0.81, 1.80)
**Occupational activity between the 8th-12th week of pregnancy**													
Yes	1.04 (0.79, 1.38)	0.97 (0.73, 1.31)	1.12 (0.87, 1.44)	0.97 (0.73, 1.28)	0.73 (0.57,0.93)^	0.79 (0.53, 1.16)	0.90 (0.71, 1.14)	1.02 (0.78, 1.32)	0.97 (0.76, 1.23)	1.07 (0.81, 1.41)	0.96 (0.74, 1.24)	0.94 (0.74, 1.19)	0.92 (0.62, 1.36)
**Socio-economic status (SES)**													
High	1.27 (0.94, 1.72)	1.40 (1.01,1.91)^	1.16 (0.88, 1.54)	0.98 (0.72, 1.35)	1.22 (0.93, 1.61)	1.11 (0.73, 1.73)	1.59 (1.22, 2.07)*	1.35 (1.01,1.79)^	1.29 (0.99, 1.69)	0.97 (0.72, 1.33)	0.94 (0.71, 1.25)	1.00 (0.77, 1.31)	0.78 (0.52, 1.20)
**Pre-pregnancy BMI (kg/m^2)**													
< 18.5	0.82 (0.49, 1.32)	0.94 (0.56, 1.53)	0.70 (0.43, 1.09)	0.90 (0.58, 1.44)	0.68 (0.46, 1.02)	0.66 (0.39, 1.19)	0.83 (0.55, 1.24)	0.79 (0.49, 1.24)	0.74 (0.48, 1.11)	0.54 (0.35,0.83)*	0.79 (0.53, 1.22)	0.92 (0.62, 1.39)	0.39 (0.23,0.67)#
≥ 25	0.96 (0.67, 1.35)	0.71 (0.47, 1.05)	0.94 (0.68, 1.28)	1.23 (0.86, 1.78)	1.01 (0.74, 1.36)	1.17 (0.73, 1.97)	1.35 (1.01, 1.81)^	0.83 (0.59, 1.16)	0.87 (0.64, 1.17)	0.80 (0.57, 1.13)	0.85 (0.63, 1.18)	0.89 (0.66, 1.20)	0.99 (0.60, 1.70)
**Leisure – time physical activity (LTPA)**													
Yes	1.58 (1.17, 2.16)*	1.58 (1.15,2.22)*	1.49 (1.13,1.97)*	1.82 (1.38,2.40)#	1.35 (1.06,1.73)^	1.66 (1.15,2.40)*	1.52 (1.19, 1.96)*	1.20 (0.91, 1.59)	1.32 (1.03,1.70)^	1.64 (1.25,2.17)#	1.19 (0.92, 1.55)	1.32 (1.03, 1.68)^	2.00 (1.36,2.92)#
**Perceived Stress Scale (range: 0-38 points)(PSS)**													
≥ 17 points	0.84 (0.64, 1.10)	0.78 (0.59, 1.04)	0.85 (0.66, 1.08)	0.75 0.57,0.98)^	0.81 (0.64, 1.02)	0.98 (0.68, 1.41)	0.67 (0.53,0.83)#	0.95 (0.74, 1.22)	0.78 (0.62,0.98)^	0.71 (0.54,0.93)^	0.78 (0.61, 0.99)	0.77 (0.61, 0.97)^	0.59 (0.40,0.87)*
**Place of residence (thousands of inhabitants)**													
< 100	0.75 (0.56,0.99)^	0.79 (0.58, 1.07)	0.82 (0.63, 1.05)	0.90 (0.69, 1.19)	0.97 (0.76, 1.23)	0.80 (0.56, 1.17)	0.86 (0.68, 1.08)	0.90 (0.69, 1.17)	1.12 (0.88, 1.42)	0.94 (0.72, 1.24)	1.00 (0.77, 1.29)	1.01 (0.80, 1.28)	0.85 (0.58, 1.25)
**Season for data collection**													
May-October	0.92 (0.71, 1.20)	0.78 (0.58, 1.04)	1.04 (0.81, 1.33)	1.22 (0.94, 1.60)	1.23 (0.98, 1.56)	0.99 (0.69, 1.42)	1.04 (0.83, 1.31)	1.09 (0.84, 1.40)	0.88 (0.70, 1.11)	1.17 (0.89, 1.52)	1.02 (0.80, 1.30)	0.99 (0.79, 1.24)	0.96 (0.66, 1.41)
**Parity**													
≥ 1	1.03 (0.79, 1.35)	1.17 (0.88, 1.55)	1.09 (0.85, 1.39)	1.39 (1.06,1.82)^	1.50 (1.19,1.89)*	1.73 (1.20,2.53)*	0.97 (0.78, 1.22)	0.98 (0.76, 1.26)	1.01 (0.81, 1.28)	1.15 (0.88, 1.50)	1.35 (1.06, 1.73)^	1.12 (0.89, 1.41)	1.35 (0.92, 1.98)
**Week of pregnancy of the 1st medical-care visit**													
> 6	0.82 (0.62,1.08)	0.89 (0.66,1.19)	0.85 (0.66,1.10)	0.81 (0.62,1.07)	0.85 (0.67,1.08)	0.71 (0.50,1.03)	0.72 (0.57,0.91)*	0.80 (0.61,1.03)	0.84 (0.66,1.07)	0.85 (0.65,1.11)	0.75 (0.59,0.96)^	0.76 (0.60,0.96)^	1.00 (0.68,1.47)

Odds ratio (OR) with 95% confidence interval (95% CI) calculated using univariable logistic regression model with the single explanatory factor

Reference groups: maternal age - ≤ 30 years; maternal education - ≤ 12 years; occupational activity between the 8th - 12th week of pregnancy - no; SES – low/medium; pre-pregnancy BMI (kg/m^2) – 18.5 – 24.99; LTPA - no; PSS - < 17 points; place of residence (thousands of inhabitants) - ≥ 100; season for data collection- November - April; parity - 0; week of pregnancy of the 1st medical-care visit - ≤ 6.

^p<0.05; *p<0.01; #p<0.001

Table S6. Univariable models for adequacy of intake of selected essential elements and vitamins from total: dietary and supplements sources (n=1005)

Determinant	Calcium	Magnesium	Iron	Zinc	Copper	Selenium	Folate	Vitamin A##	Vitamin C	Vitamin E	Vitamin B_1	Vitamin B_2	Vitamin B_3	Vitamin B_6	Vitamin B_{12}
	OR (95%CI)														
**Maternal age**															
>30	0.91 (0.69, 1.19)	1.04 (0.79, 1.35)	1.14 (0.84, 1.53)	1.07 (0.83, 1.36)	1.47 (1.09,1.99)^	1.59 (1.23, 2.08)*	1.30 (0.81, 2.14)	2.05 (1.34, 3.26)*	0.83 (0.65, 1.06)	1.01 (0.79, 1.30)	1.02 (0.80, 1.30)	1.29 (0.97, 1.73)	1.32 (0.98, 1.80)	1.05 (0.79, 1.41)	0.87 (0.54, 1.45)
**Maternal education (years of education)**															
>12	1.39 (1.04, 1.88)^	1.58 (1.18, 2.14)*	1.05 (0.77, 1.44)	1.30 (1.01,1.69)^	1.22 (0.91, 1.63)	1.26 (0.97, 1.63)	2.58 (1.65,4.03)#	1.52 (1.03,2.23)^	1.32 (1.03,1.69)^	0.91 (0.70, 1.18)	1.28 (0.99, 1.65)	1.25 (0.94, 1.66)	1.54 (1.15,2.06)*	1.22 (0.91, 1.63)	1.23 (0.74, 2.01)
**Occupational activity between the 8th-12th week of pregnancy**															
Yes	1.09 (0.83, 1.43)	1.08 (0.83, 1.41)	1.07 (0.80, 1.45)	1.22 (0.96, 1.55)	1.00 (0.75, 1.33)	0.79 (0.61, 1.01)	1.11 (0.70, 1.76)	0.83 (0.55, 1.22)	0.99 (0.78, 1.26)	1.15 (0.90, 1.48)	0.97 (0.76, 1.23)	1.04 (0.79, 1.38)	1.03 (0.77, 1.38)	1.08 (0.81, 1.44)	1.18 (0.72, 1.90)
**Socio-economic status (SES)**															
High	1.29 (0.96, 1.72)	1.26 (0.93, 1.68)	0.69 (0.47, 0.97)	1.10 (0.84, 1.44)	0.93 (0.68, 1.28)	1.07 (0.81, 1.42)	1.12 (0.67, 1.95)	1.07 (0.70, 1.68)	1.72 (1.30,2.31)#	1.32 (1.00,1.73)	1.25 (0.96, 1.63)	0.93 (0.69, 1.28)	1.06 (0.77, 1.49)	0.96 (0.70, 1.32)	1.08 (0.63, 1.93)
**Pre-pregnancy BMI (kg/m²)**															
< 18.5	0.72 (0.43, 1.16)	0.68 (0.40, 1.08)	0.62 (0.34, 1.06)	0.64 (0.41,0.98)^	0.91 (0.58, 1.47)	0.71 (0.47, 1.08)	1.06 (0.50, 2.59)	0.77 (0.43, 1.46)	0.79 (0.53, 1.19)	0.80 (0.51, 1.22)	0.78 (0.51, 1.17)	0.51 (0.33,0.78)*	1.02 (0.63, 1.73)	1.16 (0.71, 1.98)	0.50 (0.26, 1.04)
≥ 25	0.89 (0.63, 1.24)	0.74 (0.51, 1.04)	0.80 (0.54, 1.17)	0.85 (0.63, 1.16)	1.21 (0.85, 1.77)	0.94 (0.69, 1.29)	0.90 (0.52, 1.61)	1.12 (0.69, 1.89)	1.10 (0.82, 1.50)	0.79 (0.57, 1.07)	0.85 (0.63, 1.15)	0.77 (0.55, 1.10)	0.86 (0.61, 1.24)	0.81 (0.58, 1.15)	0.85 (0.47, 1.62)
**Leisure – time physical activity (LTPA)**															
Yes	1.54 (1.15, 2.08)*	1.39 (1.05, 1.87)^	1.07 (0.79, 1.47)	1.39 (1.07,1.80)^	1.74 (1.31,2.30)#	1.40 (1.08,1.80)^	1.05 (0.64, 1.67)	1.63 (1.11,2.38)^	1.25 (0.97, 1.60)	1.17 (0.90, 1.52)	1.33 (1.03,1.71)^	1.65 (1.25,2.18)#	1.28 (0.95, 1.71)	1.41 (1.05,1.88)^	2.05 (1.27, 3.30)*
**Perceived Stress Scale (range: 0-38 points)(PSS)**															
≥ 17 points	0.86 (0.66, 1.12)	0.84 (0.65, 1.09)	0.98 (0.73, 1.30)	0.94 (0.74, 1.18)	0.77 (0.59, 1.02)	0.77 (0.60,0.98)^	0.85 (0.54, 1.33)	1.05 (0.72, 1.53)	0.74 (0.58,0.93)^	0.96 (0.76, 1.22)	0.79 (0.63,1.00)^	0.73 (0.56,0.96)^	0.80 (0.60, 1.06)	0.77 (0.59, 1.02)	0.70 (0.42, 1.13)
**Place of residence (thousands of inhabitants)**															
< 100	0.72 (0.54, 0.94)^	0.79 (0.60, 1.04)	0.88 (0.65, 1.18)	0.78 (0.61,1.00)	0.89 (0.67, 1.18)	1.01 (0.78, 1.30)	1.26 (0.79, 2.07)	0.80 (0.55, 1.18)	0.94 (0.74, 1.19)	0.99 (0.77, 1.26)	1.08 (0.85, 1.37)	0.92 (0.70, 1.21)	1.00 (0.74, 1.34)	0.99 (0.74, 1.32)	0.99 (0.61, 1.64)
**Season for data collection**															
May-October	0.99 (0.76, 1.28)	0.91 (0.70, 1.18)	1.14 (0.86, 1.52)	1.14 (0.91, 1.44)	1.30 (0.99, 1.71)	1.20 (0.94, 1.52)	0.91 (0.58, 1.41)	0.99 (0.68, 1.44)	0.97 (0.77, 1.22)	1.23 (0.97, 1.55)	0.85 (0.68, 1.07)	1.16 (0.89, 1.52)	1.03 (0.78, 1.36)	0.99 (0.75, 1.31)	0.76 (0.47, 1.23)
**Parity**															
≥ 1	0.91 (0.70, 1.18)	1.10 (0.85, 1.43)	1.21 (0.91, 1.61)	1.03 (0.82, 1.30)	1.46 (1.11, 1.92)*	1.71 (1.34,2.18)#	0.89 (0.57, 1.38)	1.68 (1.16,2.48)*	0.93 (0.74, 1.17)	0.95 (0.75, 1.20)	0.96 (0.77, 1.21)	1.19 (0.91, 1.56)	1.23 (0.93, 1.63)	1.23 (0.93, 1.62)	1.05 (0.65, 1.68)
**Week of pregnancy of the 1st medical-care visit**															
> 6	0.83 (0.63, 1.08)	0.86 (0.66, 1.13)	0.97 (0.72, 1.31)	0.82 (0.64, 1.04)	0.78 (0.59, 1.03)	0.78 (0.61,0.99)^	0.53 (0.33,0.82)*	0.70 (0.48, 1.01)	0.87 (0.69, 1.11)	0.79 (0.61, 1.00)	0.87 (0.69, 1.10)	0.85 (0.65, 1.12)	0.83 (0.62, 1.10)	0.88 (0.67, 1.16)	1.07 (0.66, 1.77)

Odds ratio (OR) with 95% confidence interval (95% CI) calculated using univariable logistic regression model with the single explanatory factor

Reference groups: maternal age - ≤ 30 years; maternal education - ≤ 12 years; occupational activity between the 8th - 12th week of pregnancy - no; SES – low/medium; pre-pregnancy BMI (kg/m²) – 18.5 – 24.99; LTPA - no; PSS - < 17 points; place of residence (thousands of inhabitants) - ≥ 100; season for data collection- November - April; parity - 0; week of pregnancy of the 1st medical-care visit - ≤ 6.

^p<0.05; *p<0.01; #p<0.001

Table S7. Multivariable models for adequacy of intake of selected essential elements and vitamins from dietary sources (n=1011)

Determinant	Calcium	Magnesium	Zinc	Copper	Selenium	Vitamin A	Vitamin C	Vitamin E	Vitamin B_1	Vitamin B_2	Vitamin B_3	Vitamin B_6	Vitamin B_{12}
	OR (95%CI)												
**Maternal age**													
>30	0.94 (0.68, 1.29)	0.88 (0.62, 1.24)	1.07 (0.80, 1.43)	1.29 (0.92, 1.82)	1.44 (1.08, 1.93)^	1.53 (0.95, 2.53)	0.94 (0.71, 1.24)	1.15 (0.85, 1.55)	0.97 (0.73, 1.28)	1.09 (0.78, 1.52)	1.28 (0.94, 1.75)	0.94 (0.71, 1.24)	0.77 (0.49, 1.24)
**Maternal education (years of education)**													
>12	1.46 (1.05,2.07)^	1.58 (1.10,2.31)^	1.42 (1.05,1.93)^	1.15 (0.83, 1.58)	1.47 (1.11, 1.95)*	1.40 (0.91, 2.13)	1.29 (0.97, 1.71)	0.86 (0.64, 1.17)	1.36 (1.02, 1.81)^	1.19 (0.86, 1.63)	1.36 (1.01, 1.82)^	1.17 (0.88, 1.54)	1.26 (0.79, 1.96)
**Occupational activity between the 8th-12th week of pregnancy**													
Yes	0.99 (0.74, 1.33)	0.92 (0.67, 1.25)	1.08 (0.82, 1.41)	0.98 (0.73, 1.32)	0.68 (0.52, 0.88)*	0.73 (0.47, 1.10)	0.87 (0.68, 1.12)	1.06 (0.80, 1.40)	0.91 (0.71, 1.18)	1.02 (0.75, 1.37)	0.90 (0.69, 1.19)	0.90 (0.70, 1.16)	0.78 (0.51, 1.20)
**Socio-economic status (SES)**													
High	1.21 (0.88, 1.67)	1.34 (0.95, 1.87)	1.12 (0.83, 1.51)	1.05 (0.75, 1.48)	1.26 (0.94, 1.70)	1.25 (0.79, 2.04)	1.54 (1.16, 2.04)*	1.36 (1.00, 1.83)	1.29 (0.97, 1.70)	0.97 0.69, 1.36)	0.94 (0.69, 1.29)	0.98 (0.74, 1.31)	0.76 (0.49, 1.22)
**Pre-pregnancy BMI (kg/m²)**													
< 18.5	0.76 (0.43, 1.29)	1.01 (0.58, 1.70)	0.65 (0.38, 1.06)	0.99 (0.60, 1.69)	0.68 (0.43, 1.07)	0.71 (0.38, 1.41)	0.78 (0.49, 1.23)	0.90 (0.54, 1.46)	0.74 (0.46, 1.17)	0.53 (0.33, 0.87)*	0.78 (0.49, 1.26)	0.87 (0.56, 1.37)	0.39 (0.22, 0.71)*
≥ 25	0.90 (0.62, 1.28)	0.68 (0.44, 1.01)	0.87 (0.62, 1.21)	1.11 (0.76, 1.64)	0.94 (0.68, 1.30)	1.20 (0.72, 2.12)	1.31 (0.96, 1.78)	0.82 (0.57, 1.16)	0.87 (0.63, 1.19)	0.79 (0.55, 1.14)	0.77 (0.55, 1.07)	0.81 (0.60, 1.11)	0.98 (0.58, 1.73)
**Leisure – time physical activity (LTPA)**													
Yes	1.51 (1.10, 2.09)^	1.49 (1.06, 2.12)^	1.49 (1.12, 2.01)*	1.91 (1.42, 2.58)#	1.38 (1.05, 1.80)^	1.69 (1.12, 2.52)^	1.38 (1.06, 1.81)^	1.11 (0.83, 1.49)	1.35 (1.03, 1.77)^	1.75 (1.30, 2.35)#	1.27 (0.95, 1.68)	1.38 (1.06, 1.80)^	1.95 (1.29, 2.95)*
**Perceived Stress Scale (range: 0-38 points)(PSS)**													
≥17 points	0.97 (0.73, 1.29)	0.82 (0.61, 1.11)	0.90 (0.69, 1.16)	0.74 (0.55, 0.99)^	0.79 (0.61, 1.02)	1.04 (0.70, 1.55)	0.70 (0.55, 0.89)*	0.94 (0.72, 1.24)	0.83 (0.65, 1.07)	0.75 (0.56, 1.00)	0.76 (0.58, 0.99)^	0.76 (0.59, 0.97)^	0.58 (0.38, 0.89)^
**Place of residence (thousands of inhabitants)**													
< 100	0.79 (0.58, 1.06)	0.82 (0.59, 1.13)	0.87 (0.66, 1.14)	0.93 (0.69, 1.26)	1.00 (0.77, 1.31)	0.78 (0.52, 1.17)	0.89 (0.69, 1.16)	0.88 (0.66, 1.17)	1.12 (0.86, 1.45)	0.92 (0.68, 1.24)	1.03 (0.78, 1.36)	1.02 (0.79, 1.32)	0.81 (0.53, 1.24)
**Season for data collection**													
May-October	0.98 (0.74, 1.29)	0.88 (0.65, 1.19)	1.10 (0.85, 1.43)	1.13 (0.84, 1.51)	1.21 (0.94, 1.56)	0.86 (0.58, 1.27)	1.07 (0.84, 1.36)	1.16 (0.89, 1.52)	0.97 (0.76, 1.23)	1.17 (0.88, 1.56)	0.98 (0.75, 1.28)	1.00 (0.78, 1.27)	0.87 (0.58, 1.31)
**Parity**													
≥ 1	1.11 (0.81, 1.50)	1.28 (0.92, 1.77)	1.16 (0.88, 1.54)	1.54 (1.12, 2.13)*	1.52 (1.15, 2.00)*	1.65 (1.07, 2.58)^	1.01 (0.78, 1.32)	1.05 (0.78, 1.40)	1.11 (0.85, 1.45)	1.23 (0.90, 1.69)	1.33 (1.00, 1.78)	1.24 (0.95, 1.63)	1.45 (0.93, 2.29)
**Week of pregnancy of the 1st medical-care visit**													
> 6	0.86 (0.64, 1.16)	0.91 (0.67, 1.25)	0.85 (0.65, 1.11)	0.84 (0.62, 1.13)	0.84 (0.65, 1.09)	0.66 (0.45, 0.99)^	0.77 (0.60, 0.99)^	0.82 (0.62, 1.08)	0.89 (0.69, 1.15)	0.76 (0.57, 1.02)	0.72 (0.55, 0.95)^	0.75 (0.58, 0.97)^	0.93 (0.61, 1.42)

Odds ratio (OR) with 95% confidence interval (95% CI) calculated using multivariable logistic regression model

Reference groups: maternal age - ≤ 30 years; maternal education - ≤ 12 years; occupational activity between the 8th - 12th week of pregnancy - no; SES – low/medium; pre-pregnancy BMI (kg/m²) – 18.5 – 24.99; LTPA - no; PSS - < 17 points; place of residence (thousands of inhabitants) - ≥ 100; season for data collection- November - April; parity - 0; week of pregnancy of the 1st medical-care visit - ≤ 6.

^p<0.05; *p<0.01; #p<0.001

Table S8. Multivariable models for adequacy of intake of selected essential elements and vitamins from total: dietary and supplements (n=1005)

Determinant	Calcium	Magnesium	Iron	Zinc	Copper	Selenium	Folate	Vitamin A##	Vitamin C	Vitamin E	Vitamin B_1	Vitamin B_2	Vitamin B_3	Vitamin B_6	Vitamin B_{12}
	OR (95%CI)														
**Maternal age**															
>30	0.86 (0.63, 1.18)	0.88 (0.64,1.19)	1.07 (0.76, 1.49)	0.95 (0.72, 1.26)	1.21 (0.86, 1.71)	1.29 (0.95, 1.75)	1.44 (0.83, 2.57)	1.58 (0.97, 2.66)	0.80 (0.60, 1.06)	1.03 (0.77, 1.37)	1.00 (0.76, 1.32)	1.07 (0.77, 1.50)	1.08 (0.76, 1.53)	0.86 (0.62, 1.21)	0.70 (0.40, 1.25)
**Maternal education (years of education)**															
>12	1.41 (1.02,1.96)^	1.42 (1.03, 1.97)^	0.94 (0.67, 1.33)	1.24 (0.93, 1.65)	1.10 (0.78, 1.52)	1.29 (0.96, 1.73)	2.81 (1.71,4.64)#	1.32 (0.85, 2.04)	1.26 (0.95, 1.66)	0.83 (0.62, 1.10)	1.34 (1.01, 1.78)^	1.22 (0.88, 1.68)	1.46 (1.05, 2.03)^	1.21 (0.87, 1.68)	1.44 (0.82, 2.49)
**Occupational activity between the 8th-12th week of pregnancy**															
Yes	1.02 (0.77, 1.36)	1.02 (0.77, 1.35)	1.14 (0.83, 1.57)	1.22 (0.95, 1.58)	1.05 (0.77, 1.43)	0.78 (0.59, 1.03)	1.00 (0.61, 1.64)	0.78 (0.50, 1.19)	0.95 (0.73, 1.23)	1.23 (0.94, 1.60)	0.92 (0.71, 1.18)	0.98 (0.73, 1.33)	0.94 (0.69, 1.29)	1.05 (0.77, 1.43)	1.00 (0.59, 1.68)
**Socio-economic status (SES)**															
High	1.24 (0.91, 1.69)	1.21 (0.89, 1.65)	0.69 (0.47, 1.00)	1.04 (0.78, 1.39)	1.00 (0.71, 1.42)	1.05 (0.77, 1.43)	1.13 (0.64, 2.13)	1.19 (0.74, 1.95)	1.62 (1.20,2.21)*	1.33 (1.00, 1.78)	1.24 (0.94, 1.65)	0.92 (0.66, 1.30)	1.04 (0.73, 1.50)	0.85 (0.61, 1.20)	0.96 (0.54, 1.79)
**Pre-pregnancy BMI (kg/m²)**															
< 18.5	0.66 (0.37, 1.12)	0.71 (0.41, 1.19)	0.69 (0.36, 1.23)	0.60 (0.36, 0.97)^	1.00 (0.60, 1.75)	0.72 (0.45, 1.16)	0.88 (0.39, 2.39)	0.88 (0.45, 1.90)	0.71 (0.46, 1.12)	0.88 (0.55, 1.40)	0.75 (0.47, 1.18)	0.49 (0.31, 0.80)*	0.91 (0.53, 1.62)	1.07 (0.62, 1.93)	0.38 (0.19, 0.81)*
≥ 25	0.82 (0.57, 1.17)	0.72 (0.50, 1.03)	0.80 (0.53, 1.17)	0.81 (0.59, 1.11)	1.10 (0.75, 1.63)	0.86 (0.62, 1.20)	0.83 (0.47, 1.55)	1.14 (0.67, 2.01)	1.07 (0.78, 1.48)	0.79 (0.57, 1.10)	0.85 (0.62, 1.17)	0.76 (0.53, 1.10)	0.79 (0.55, 1.15)	0.75 (0.53, 1.09)	0.89 (0.47, 1.81)
**Leisure – time physical activity (LTPA)**															
Yes	1.45 (1.06,1.98)^	1.30 (0.96, 1.77)	1.08 (0.78, 1.51)	1.37 (1.04, 1.81)^	1.80 (1.32,2.44)#	1.46 (1.10,1.93)*	1.22 (0.72, 2.02)	1.66 (1.09,2.50)^	1.14 (0.87, 1.49)	1.06 (0.81, 1.40)	1.36 (1.04, 1.78)^	1.75 (1.29, 2.36)#	1.31 (0.95, 1.80)	1.44 (1.05, 1.96)^	1.95 (1.16, 3.25)^
**Perceived Stress Scale (range: 0-38 points)(PSS)**															
≥17 points	0.95 (0.72, 1.25)	0.88 (0.67, 1.16)	0.92 (0.68, 1.25)	0.97 (0.75, 1.24)	0.74 (0.55, 1.00)	0.73 (0.56,0.96)^	0.97 (0.60, 1.58)	1.12 (0.75, 1.68)	0.77 (0.60,0.99)^	0.94 (0.73, 1.22)	0.85 (0.66, 1.08)	0.75 (0.56, 1.01)	0.77 (0.57, 1.05)	0.72 (0.53, 0.97)^	0.74 (0.44, 1.24)
**Place of residence (thousands of inhabitants)**															
< 100	0.77 (0.57, 1.04)	0.81 (0.61, 1.09)	0.88 (0.63, 1.21)	0.82 (0.63, 1.06)	0.92 (0.68, 1.26)	1.01 (0.76, 1.33)	1.37 (0.82, 2.34)	0.78 (0.51, 1.18)	1.02 (0.78, 1.33)	0.97 (0.74, 1.27)	1.12 (0.87, 1.45)	0.91 (0.67, 1.23)	1.06 (0.77, 1.47)	1.01 (0.74, 1.38)	0.95 (0.57, 1.64)
**Season for data collection**															
May-October	1.01 (0.77, 1.34)	0.94 (0.72, 1.24)	1.06 (0.79, 1.43)	1.14 (0.89, 1.46)	1.17 (0.87, 1.57)	1.13 (0.87, 1.48)	0.93 (0.58, 1.51)	0.86 (0.57, 1.28)	0.97 (0.75, 1.24)	1.25 (0.97, 1.61)	0.91 (0.71, 1.16)	1.13 (0.85, 1.51)	0.99 (0.74, 1.34)	0.97 (0.73, 1.31)	0.71 (0.43, 1.18)
**Parity**															
≥ 1	1.03 (0.76, 1.39)	1.20 (0.89, 1.62)	1.28 (0.92, 1.79)	1.15 (0.88, 1.51)	1.65 (1.19,2.29)*	1.84 (1.38,2.46)#	0.70 (0.41, 1.18)	1.57 (1.00, 2.48)	1.03 (0.79, 1.36)	1.04 (0.79, 1.37)	1.03 (0.79, 1.35)	1.26 (0.92, 1.74)	1.34 (0.96, 1.87)	1.41 (1.02, 1.95)^	1.23 (0.70, 2.17)
**Week of pregnancy of the 1st medical-care visit**															
> 6	0.87 (0.65, 1.15)	0.87 (0.65, 1.16)	0.91 (0.66, 1.24)	0.80 (0.62, 1.03)	0.81 (0.60, 1.10)	0.74 (0.56,0.97)^	0.52 (0.31,0.84)*	0.66 (0.43,0.99)^	0.93 (0.72, 1.20)	0.78 (0.60, 1.01)	0.93 (0.72, 1.19)	0.77 (0.57, 1.04)	0.77 (0.56, 1.04)	0.80 (0.59, 1.08)	0.89 (0.53, 1.52)

Odds ratio (OR) with 95% confidence interval (95% CI) calculated using multivariable logistic regression model

Reference groups: maternal age - ≤ 30 years; maternal education - ≤ 12 years; occupational activity between the 8th - 12th week of pregnancy - no; SES – low/medium; pre-pregnancy BMI (kg/m^2) – 18.5 – 24.99; LTPA - no; PSS - < 17 points; place of residence (thousands of inhabitants) - ≥ 100; season for data collection- November - April; parity - 0; week of pregnancy of the 1st medical-care visit - ≤ 6. ##N=1003

^p<0.05; *p<0.01; #p<0.001

Table S9. Microelements and vitamins concentrations in plasma collected during the 1st trimester of pregnancy (microelements: n=340; vitamins: n=358)

Determinant	Zinc (mg/l)	Copper (mg/l)	Selenium (μg/l)	Vitamin A (mg/l)	Vitamin E (mg/l)
			Mean (SD)		
**Maternal age (years)**					
≤ 30	0.89 (0.26)	1.96 (0.57)	46.77 (10.84)	0.97 (0.28)	7.74 (3.50)
>30	0.93 (0.28)	1.98 (0.59)	49.84 (10.35)^	0.99 (0.29)	9.09 (3.49)*
**Maternal education (years of education)**					
≤ 12	0.95 (0.23)	1.97 (0.54)	47.53 (11.49)	0.97 (0.31)	8.35 (3.45)
>12	0.89 (0.28)	1.97 (0.59)	48.12 (10.50)	0.98 (0.27)	8.12 (3.60)
**Occupational activity between the 8th-12th week of pregnancy**					
No	0.96 (0.28)	2.05 (0.51)	47.32 (10.96)	0.91 (0.26)	8.41 (3.72)
Yes	0.87 (0.25)*	1.94 (0.61)	48.31 (10.64)	1.01 (0.29)*	8.13 (3.40)
**Socio-economic status (SES)**					
Low/medium	0.91 (0.26)	1.98 (0.59)	47.09 (10.69)	0.97 (0.28)	8.31 (3.62)
High	0.91 (0.29)	1.93 (0.53)	50.51 (10.05)^	0.99 (0.28)	7.87 (3.42)
**Pre-pregnancy BMI (kg/m^2)**					
< 18.5	0.91 (0.27)	1.79 (0.52)	46.20 (8.35)	0.89 (0.27)	7.12 (4.42)
18.5 -24.99	0.89 (0.24)	1.94 (0.54)	47.46 (10.03)	0.97 (0.27)	8.25 (3.38)
≥ 25	0.96 (0.35)	2.14 (0.68)^	50.23 (13.77)	1.02 (0.34)	8.36 (3.88)
**Cotinine level**					
< 10 ng/ml	0.90 (0.27)	1.96 (0.58)	48.22 (10.70)	0.97 (0.28)	8.11 (3.55)
≥ 10 ng/ml	0.96 (0.22)	2.01 (0.50)	45.43 (10.80)	1.00 (0.29)	8.97 (3.44)
**Alcohol consumption**					
No	0.90 (0.27)	1.98 (0.58)	48.10 (10.97)	0.96 (0.28)	8.07 (3.54)
Yes	0.94 (0.23)	1.97 (0.53)	46.39 (8.76)	1.08 (0.34)^	9.44 (3.53)
**Leisure – time physical activity (LTPA)**					
No	0.87 (0.24)	1.95 (0.53)	46.41 (8.47)	0.94 (0.28)	8.34 (3.52)
Yes	0.92 (0.28)	1.98 (0.60)	48.62 (11.53)	0.98 (0.28)	8.12 (3.56)
**Perceived Stress Scale (range: 0-38 points) (PSS)**					
< 17 points	0.87 (0.25)	1.98 (0.58)	48.59 (10.92)	0.99 (0.28)	7.94 (3.69)
≥ 17 points	0.93 (0.28)^	1.96 (0.57)	47.58 (10.51)	0.95 (0.28)	8.46 (3.37)
**Place of residence (thousands of inhabitants)**					
< 100	0.85 (0.21)^	2.04 (0.61)	48.47 (11.30)	0.91 (0.29)*	8.17 (3.42)
≥ 100	0.92 (0.28)	1.95 (0.56)	47.76 (10.54)	1.00 (0.28)	8.20 (3.61)
**Season for data collection**					
November-April	0.90 (0.29)	1.93 (0.62)	46.46 (11.72)	0.99 (0.27)	7.73 (3.70)
May-October	0.91 (0.25)	2.00 (0.53)	49.17 (9.69)^	0.96 (0.29)	8.66 (3.33)^
**Parity**					
0	0.89 (0.24)	1.90 (0.52)	47.35 (10.86)	0.97 (0.28)	8.23 (3.62)
≥ 1	0.92 (0.30)	2.05 (0.63)^	48.65 (10.62)	0.98 (0.29)	8.12 (3.49)
**Week of pregnancy of the 1st medical-care**					
≤ 6	0.90 (0.28)	1.98 (0.61)	47.94 (11.22)	0.96 (0.29)	8.35 (3.67)
> 6	0.92 (0.25)	1.97 (0.53)	47.99 (10.13)	1.00 (0.28)	7.99 (3.40)

Table S10. Coefficients of univariable linear regression model of microelements and vitamins concentration in plasma collected during the 1st trimester of pregnancy depending on selected factors (microelements: n=340; vitamins: n=358)

Determinant	Zinc (mg/l)	Copper (mg/l)	Selenium (μg/l)	Vitamin A (mg/l)	Vitamin E (mg/l)
			β (95%CI)		
**Maternal age (years)**					
>30	0.04 (-0.02, 0.10)	0.02 (-0.11, 0.15)	3.07 (0.73, 5.41)^	0.02 (-0.04, 0.08)	1.35 (0.58, 2.11)*
**Maternal education (years of education)**					
> 12	-0.06 (-0.12, 0.01)	0.00 (-0.14, 0.14)	0.60 (-1.99, 3.18)	0.01 (-0.06, 0.08)	-0.23 (-1.08, 0.62)
**Occupational activity between the 8th-12th week of pregnancy**					
Yes	-0.08 (-0.14, -0.02)*	-0.11 (-0.24, 0.01)	0.99 (-1.40, 3.39)	0.10 (0.04, 0.17)*	-0.28 (-1.06, 0.50)
**Socio-economic status (SES)**					
High	0.00 (-0.06, 0.07)	-0.06 (-0.20, 0.09)	3.42 (0.71, 6.13)^	0.02 (-0.04, 0.09)	-0.44 (-1.28, 0.41)
**Pre-pregnancy BMI (kg/m^2)**					
< 18.5	0.02 (-0.08, 0.13)	-0.14 (-0.37, 0.08)	-1.25 (-5.48, 2.97)	-0.08 (-0.20, 0.03)	-1.13 (-2.62, 0.36)
≥ 25	0.07 (0.00, 0.15)	0.20 (0.05, 0.36)^	2.77 (-0.17, 5.71)	0.05 (-0.02, 0.13)	0.11 (-0.86, 1.08)
**Cotinine level**					
≥ 10ng/ml	0.06 (-0.04, 0.15)	0.05 (-0.16, 0.25)	-2.80 (-6.60, 1.01)	0.03 (-0.07, 0.13)	0.87 (-0.37, 2.11)
**Alcohol consumption**					
Yes	0.04 (-0.06, 0.14)	-0.00 (-0.21, 0.21)	-1.71 (-5.65, 2.23)	0.11 (0.01, 0.22)^	1.37 (0.02, 2.72)
**Leisure – time physical activity (LTPA)**					
Yes	0.05 (-0.01, 0.12)	0.03 (-0.10, 0.17)	2.22 (-0.26, 4.69)	0.04 (-0.02, 0.10)	-0.22 (-1.02, 0.58)
**Perceived Stress Scale (rang: 0-38 points) (PSS)**					
≥ 17 points	0.06 (0.01, 0.12)^	-0.03 (-0.15, 0.10)	-1.01 (-3.31, 1.29)	-0.03 (-0.09, 0.02)	0.52 (-0.22, 1.25)
**Place of residence (thousands of inhabitants)**					
< 100	-0.07 (-0.13, -0.00)^	0.09 (-0.05, 0.23)	0.71 (-1.91, 3.34)	-0.09(-0.15, -0.03)*	-0.02 (-0.83, 0.79)
**Season for data collection**					
May-October	0.00 (-0.05, 0.06)	0.07 (-0.05, 0.19)	2.71 (0.43, 5.00)^	-0.03 (-0.09, 0.03)	0.93 (0.19, 1.66)^
**Parity**					
≥ 1	0.03 (-0.03, 0.09)	0.14 (0.02, 0.27)^	1.30 (-1.01, 3.61)	0.00 (-0.06, 0.06)	-0.11 (-0.86, 0.63)
**Week of pregnancy of the 1st medical-care visit**					
> 6	0.01 (-0.04, 0.07)	-0.01 (-0.13, 0.12)	0.05 (-2.33, 2.43)	0.04 (-0.02, 0.10)	-0.36 (-1.12, 0.41)

Effect of each determinant is presented as multiple regression coefficient β with 95% confidence intervals (95%CI) and represent adjusted change of concentration with respect to respective reference group.
Reference groups: maternal age - ≤ 30 years; maternal education - ≤ 12 years; occupational activity between the 8th - 12th week of pregnancy - no; SES – low/medium; pre-pregnancy BMI (kg/m^2) – 18.5 – 24.99; cotinine level - < 10 ng/ml; alcohol consumption - no; LTPA - no; PSS - <17 points; place of residence (thousands of inhabitants) - ≥100; season - November - April; parity - 0; week of pregnancy of the 1st medical-care visit - ≤6.
^p<0.05; *p<0.01
